# Identification and investigation of depression-related molecular subtypes in inflammatory bowel disease and the anti-inflammatory mechanisms of paroxetine

**DOI:** 10.3389/fimmu.2023.1145070

**Published:** 2023-02-27

**Authors:** Lijun Ning, Xinyuan Wang, Baoqin Xuan, Yanru Ma, Yuqing Yan, Ziyun Gao, Tianying Tong, Zhe Cui, Haoyan Chen, Xiaobo Li, Jie Hong, Zhenhua Wang

**Affiliations:** ^1^ State Key Laboratory for Oncogenes and Related Genes, Key Laboratory of Gastroenterology and Hepatology, Ministry of Health, Division of Gastroenterology and Hepatology, Shanghai Cancer Institute, Shanghai Institute of Digestive Disease, Renji Hospital, Shanghai Jiao Tong University School of Medicine, Shanghai, China; ^2^ Department of Gastrointestinal Surgery, Renji Hospital, Shanghai Jiao Tong University School of Medicine, Shanghai, China

**Keywords:** inflammatory bowel disease, depression, non-negative matrix factorization, antidepressants, paroxetine

## Abstract

**Background:**

Up to 40 per cent of people with active inflammatory bowel disease (IBD) also suffer from mood disorders such as anxiety and depression. Notwithstanding, the fundamental biological pathways driving depression in IBD remain unknown.

**Methods:**

We identified 33 core genes that drive depression in IBD patients and performed consensus molecular subtyping with the NMF algorithm in IBD. The CIBERSORT were employed to quantify the immune cells. Metabolic signature was characterized using the “IOBR” R package. The scoring system (D. score) based on PCA. Pre-clinical models are constructed using DSS.

**Results:**

Using transcriptome data from the GEO database of 630 IBD patients, we performed a thorough analysis of the correlation between IBD and depression in this research. Firstly, the samples were separated into two different molecular subtypes (D. cluster1 and D. cluster2) based on their biological signatures. Moreover, the immunological and metabolic differences between them were evaluated, and we discovered that D. cluster2 most closely resembled IBD patients concomitant with depression. We also developed a scoring system to assess the IBD-related depression and predict clinical response to anti-TNF- therapy, with a higher D. score suggesting more inflammation and worse reaction to biological therapies. Ultimately, we also identified through animal experiments an antidepressant, paroxetine, has the added benefit of lowering intestinal inflammation by controlling microorganisms in the digestive tract.

**Conclusions:**

This study highlights that IBD patients with or without depression show significant variations and antidepressant paroxetine may help reduce intestinal inflammation.

## Introduction

Inflammatory bowel disease (IBD), which includes Crohn’s disease (CD) and ulcerative colitis (UC), is considered to be caused by an abnormal immune response to enteric microbiota and environmental triggers in a genetically vulnerable host, and its incidence and prevalence are growing globally (1, 2). IBD is a severe gastrointestinal illness that causes symptoms like abdominal pain and feculent blood, as well as a lower quality of life and deficiencies in social roles, which may put people with IBD at a higher risk of depression and anxiety than the general population (3–6). Notably, active IBD is connected with the occurrence of anxiety and sadness in up to 40% of patients (7). Nevertheless, the molecular biological mechanisms underlying such IBD patients combined with depression are unclear thus far.

Given the strong genetic and hereditary correlates between IBD and depression (8–10), recognizing the mechanism that links IBD to depression and anxiousness is essential for designing therapeutic and prevention measures, as well as predicting prognosis in IBD patients properly. Over the last two decades, there has been a wealth of research linking depression and anxiety to systemic immunological engagement, which includes abnormalities in inflammatory mediators, immune cell populations, and antibody titers (11, 12). A study on neuroendocrine-immune interactions in patients with major depressive disorder(MDD) found that the pro-inflammatory cytokine TNF-α was significantly higher in the serum of MDD patients than in normal controls, confirming increased inflammation and dysregulation of the immune system in MDD patients (13). Similarly, immunopathological processes like macrophage polarization and neutrophil infiltration are also present in IBD. As IBD involves aberrant activation of innate and adaptive immune responses (14), immune dysfunction may be a common pathway between it and depression. Moreover, recent studies have found that the metabolic disorder is another mechanism that shadows the onset of IBD and the state of depression. Researchers discovered that elevated levels of tryptophan metabolites suggest a high activity of tryptophan degradation in persons with active IBD after investigating over 500 IBD patients (15)., which means tryptophan deficiency could contribute to the development of IBD or aggravate disease activity. Another study showed that disturbances of the kynurenine metabolic pathway, one of the metabolic pathways of tryptophan, collectively contribute to the development of depression-like behaviour (16). Therefore, the relationship between IBD and depression may be examined from the perspective of immune activation and metabolic dysfunction.

In addition, as a common gut-brain therapy, antidepressants tend to be beneficial not only in treating mood disorders but also in relieving discomfort and reducing the risk of relapse in patients with IBD (17), although data remains limited. Antidepressants have been demonstrated in certain studies to aid with comorbid functional problems, regulate chronic diarrhea during IBD remission, and maybe decrease inflammation and improve disease activity (17). Furthermore, given the immunomodulatory effects of serotonin and its reuptake inhibitors (18, 19), we hypothesize that SSRI-type antidepressants may play an essential role in modulating the immune-inflammation. Hence, it is essential to explore how antidepressants alleviate intestinal inflammation.

As sequencing technology advances, a growing amount of transcriptomic data become available in public databases, offering a handy tool for a thorough investigation into the mechanisms underlying both IBD and depression. In this study, we integrated the transcriptomic data of 630 IBD samples from Gene Expression Omnibus (GEO) databases for subsequent analysis. Firstly, we utilized consensus clustering analysis of the Non-negative Matrix Factorization (NMF) algorithm to stratify samples with qualitatively different molecular subtypes. We then explored the differences between the two subtypes through a series of bioinformatic approaches from an immunological and metabolic perspective, respectively, and one of the above subtypes was considered to be IBD combined with depression. Moreover, a scoring scheme was constructed to quantify the IBD-related depression gene signature and anticipate the therapeutic outcome of patients to anti-TNF-alpha medication. We eventually discovered an antidepressant, paroxetine, that can alleviate intestinal inflammation, and its anti-inflammatory mechanism works by regulating gut microorganisms.

## Materials and methods

### Obtaining and preprocessing IBD datasets

The Gene Expression Omnibus (GEO) database (https://www.ncbi.nlm.nih.gov/geo/) is a collection of publicly available gene expression data and associated clinical information. This study recruited three IBD cohorts (GSE87473, GSE92415, and GSE112366) on the same platform for future investigation (**Table S1**). To adjust for batch effects in the various cohort datasets, the “ComBat” method was implemented in the “sva” R package. Using the “limma” R package using the criterion p. adjust 0.05 and | Log2FC | > 0.2, we were able to find the DEGs that are significantly different in expression between IBD patients and healthy controls.

### Consensus molecular clustering of 33 core genes that drive depression in IBD patients by NMF

Our NMF consensus clustering was based on the expression levels of the 33 central genes, allowing us to isolate distinct molecular subtypes. Concretely, the expression levels of 33 core genes that drive depression in IBD patients (Matrix A) were decomposed into two nonnegative matrices W and H (i.e., A≈WH). The outputs of Matrix A were aggregated after repeated factorization to generate consensus clustering of IBD samples. The consensus clustering was carried out using the R package ‘NMF’ (version 0.30.4) and the brunet method. The best number of clusters was optimized using cophenetic, dispersion, and silhouette parameters.

### Gene set variation analysis (GSVA) and functional annotation

Using the “h.all.v7.5.1.symbols.gmt” gene set obtained from the “MSigDB” database, we did a GSVA enrichment analysis (“GSVA” R packages) to describe the biological processes different across depression-related IBD molecular subtypes. Statistical significance was assumed when the p-value was less than 0.0001. Functional annotation was carried out with the help of the “clusterProfiler” R package (version 4.2.0).

### Evaluation of immune infiltration levels and metabolism gene signatures

The proportion of infiltration of the 22 immune cells was assessed by the “CIBERSORT” R package (20). The activation of metabolic gene signature was analyzed using the “IOBR” R package (21). (https://github.com/IOBR/IOBR).

### Construction of the IBD-related depression gene signature scores

We developed an IBD-related depression gene signature scoring scheme (D. score) using principal component analysis (PCA). Principal components 1 and 2 of the PCA analysis based on the DEGs were retrieved and served as the signature score. We then adopted the mathematical formula (22, 23) to calculate the D. score: D. score = ∑(PC1_i_+PC2_i_).

### Mice, models and treatments

**Figure 1:**
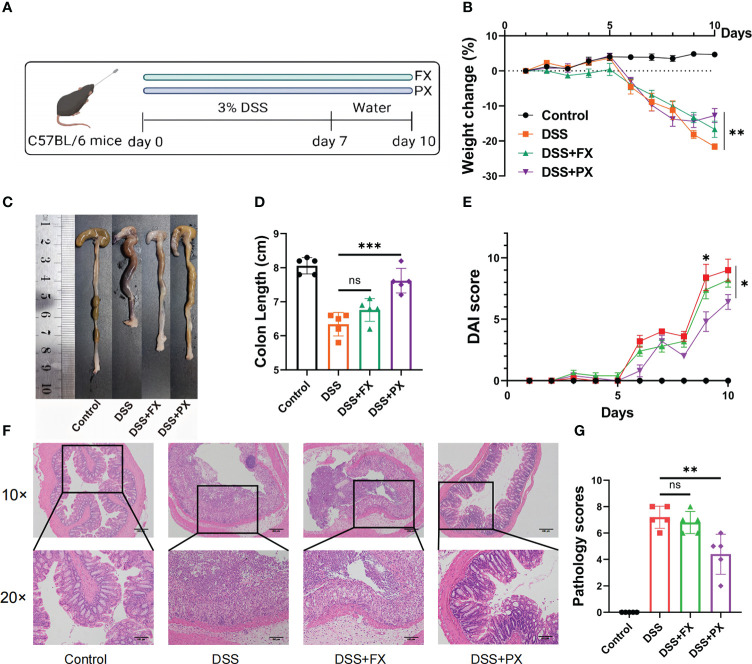
Antidepressants can resolve intestinal inflammation in a preclinical model of (D) cluster2 patients. **(A)** Induction of experimental ulcerative colitis. Mice received 3% (wt/vol) DSS dissolved in drinking water for 7 days followed by normal drinking water for 3 days. **(B)** Body weight change. **(C, D)** Representative pictures of colon gross appearance and colon length. **(E)** DAI score. **(F, G)** Representative microscopic pictures of H&E staining (40× and 100× magnification) and histology scores. Data are pooled from each independent experiment with n = 5 mice per group; The asterisks represented the statistical P-value (ns P > 0.05; *P < 0.05; **P < 0.01; ***P < 0.001).

The 6-8-week-old male C57BL/6 mice were housed and reared in specific pathogen-free conditions, at the animal center of Renji Hospital affiliated with Shanghai Jiao Tong University School of Medicine. For the DSS-induced colitis model, the mice were administered 3% DSS (molecular weight, 36,000–50,000 Da; MP Biomedicals) in drinking water for seven days, followed by three days of DSS-free water. In addition, the Disease Activity Index (DAI), a score that reflects the severity of the disease, were recorded daily, including diarrhea, bloody stools and body weight (24). After 10% formalin and paraffin embedding, mice colonic tissues were collected and stained with H&E. The pathology scores were calculated from two parameters for a maximum score of 8; one parameter is cell infiltrate (normal, 0; mild 5 mucosa, 1; moderate in mucosa, 2; marked in mucosa, 3; moderate/severe in mucosa and submucosa, 4; transmural, 5), and another architecture (no erosion, 0; focal erosion, 1; focal ulceration, 2; extended ulcerations, 3) (25). Paroxetine (Med Chem Express, USA) and Fluoxetine (Med Chem Express, USA) was suspended in phosphate buffer saline (20 mg/kg/d), and mice were given that suspension intragastrically for ten days until the end of the experiment (**Figure 1A**).

### RNA sequencing analysis

After the mice were killed, their colons were removed and frozen at -80 degrees Celsius. Trizol reagent was used to extract and purify the total RNA from each sample (Invitrogen, USA). NanoDrop 2000 was used for analyzing the concentration and purity of RNA (NanoDrop, USA). We utilized 1 ng of RNA in each sample as the starting point. Following the manufacturer’s instructions, sequencing libraries were prepared by performing steps such as mRNA purification, fragmentation, cDNA synthesis, adaptor ligation, library purification, and library amplification using an Illumina TruseqTM RNA sample prep Kit. Following that, an Agilent Bioanalyzer 2100 was used to assess the library’s quality, and a Qubit 2.0 Fluorometer was used to quantify the results (Invitrogen, Carlsbad, USA). Following the manufacturer’s recommendation, we carried out the paired-end sequencing using an Illumina HiSeq 2000.

### DNA extraction and 16S-rRNA sequencing

E.Z.N.A.Soil DNA Kit (Omega, M5635-02, USA) was used to extract genomic DNA from mouse feces following the manufacturer’s instructions. Each PCR product’s DNA concentration was measured using a Qubit^®^ 4.0 Green double-stranded DNA test (ThermoFisher, USA). Illumina HiSeq sequencing technology was utilized to sequence the library. Valid data were obtained after first stitching and filtering raw data. By measuring the abundance of OTUs, we were able to calculate -diversity indices. Principal coordinate analysis was used to illustrate variety (PCoA). LefSe’s difference comparison was used to find characteristics whose abundances varied greatly between categories. Analysis of functional predictions made by the program “Tax4Fun” was used to infer the metabolic roles of bacteria.

### Reverse-transcription and real-time PCR

The intestinal tissues of mice were processed with the Trizol reagent to get total RNA (Invitrogen, USA). In each of the samples, 1 ng of total RNA was used for the process of reverse transcription, and the PrimeScriptTM RT Master Mix was used (Takara). After that, quantitative RT-PCR was carried out in triplicates by using the StepOnePlus real-time PCR machine in conjunction with the TB Green PCR Master Mix reagent from Takara as the detector (Applied Biosystems). Gapdh was considered an internal control for mRNA expression. The relative expression levels of Grk2 and Slc6a4 were quantified using the 2^−ΔΔ^Ct method.

The following primers were used for real-time PCR analysis:

Gapdh-F, 5’- CATCACTGCCACCCAGAAGACTG-3’,
Gapdh-R, 5’- ATGCCAGTGAGCTTCCCGTTCAG -3’;
Grk2-F, 5’-CGGAAAGCAGACACAGGCAAGA-3’,
Ggk2-R, 5’-AGCATGATCCGCTCGTTCAGAG -3’;
S1c6a4-F, 5’- GTTGATGCTGCGGCTCAGATCT-3’,
S1c6a4-R, 5’- GAAGCTCGTCATGCAGTTCACC-3’.

### Statistical analyses

All of the data was analyzed and visualized using R-4.1.1 and GraphPad Prism 8.4, using Student’s t-tests for comparisons between two groups and one-way analysis of variance (ANOVA) for comparisons across multiple groups. The findings are presented in the form of the mean together with the standard deviation (SD). Not significant (NS) indicates that no statistically significant difference was found between the groups. (*p 0.05, **p 0.01, ***p 0.001, ****p 0.0001)

## Results

### Identification of two molecular subtypes by 33 genes that drive depression in IBD patients

**Figure 2:**
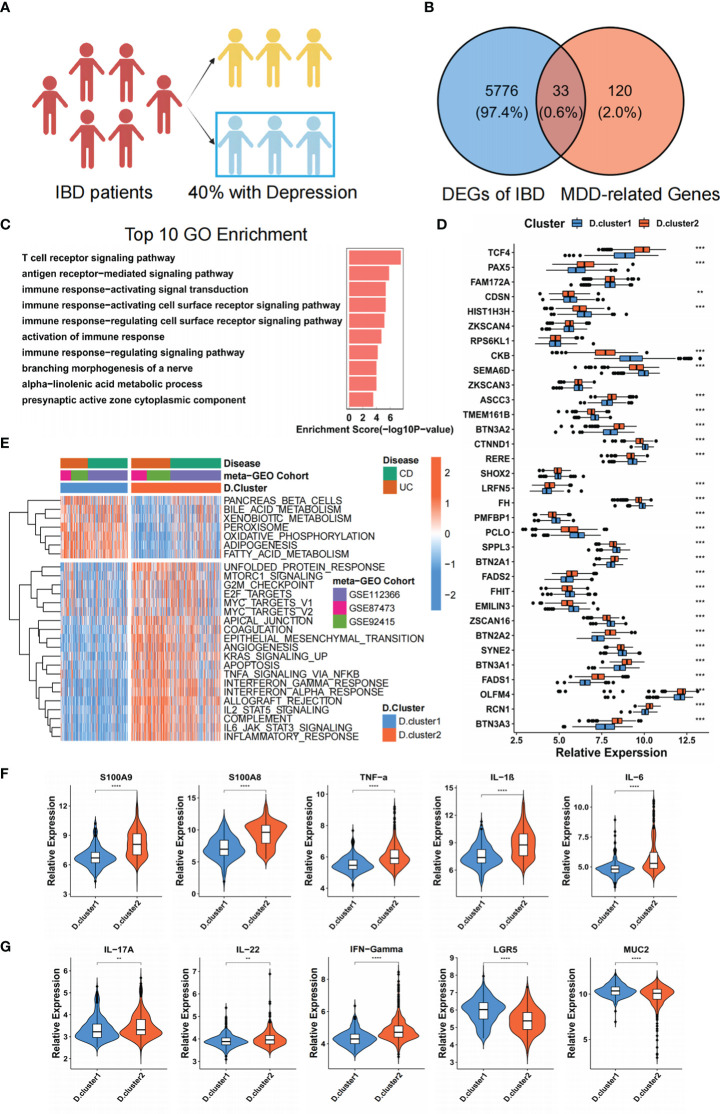
Identification of two molecular subtypes by 33 genes that drive depression in IBD patients. **(A)** The schematic representation of the proportion of IBD patients with or without depression. **(B)** The 33 core genes that might drive depression in IBD patients were shown in the Venn diagram. **(C)** Functional annotation for 33 core genes using GO enrichment analysis. **(D)** The difference in mRNA expression levels of 33 core genes between two molecular subtypes. **(E)** Heatmap shows the GSVA score of representative Hallmark pathways curated from MSigDB in distinct molecular subtypes. The GEO cohort composition (GSE87473, GSE92415, GSE112366) were used as sample annotations. **(F, G)** The mRNA expression levels of intestinal inflammatory markers (S100A8, S100A9, TNF-α, IL-1B, IL-6, IL-17, IL-22, INF-γ) and barrier function markers (LGR5, MUC2) between two molecular subtypes. The asterisks represented the statistical P-value (**P < 0.01; ***P < 0.001; ****P < 0.0001).

According to compiled data from published reports (26), up to forty per cent of individuals with active IBD also suffer from anxiety and despair (**Figure 2A**). We firstly identified potential target genes that drive depression in IBD patients. Three GEO datasets (IBD) with available clinical information (GSE87473, GSE92415, GSE112366) were enrolled into one meta-cohort. And then, we intersected DEGs with previously reported 153 depression-associated genes (27) and eventually obtained 33 genes (termed “33 core genes” hereafter) that might drive depression in IBD patients (**Figures 2B**, **S1A**). The above 33 core genes were significantly enriched in immune and metabolic-related pathways by GO enrichment analyses (**Figures 2C**, **S1B**). To further investigate transcriptome features, we used the NMF algorithm’s consensus clustering analysis to classify samples into qualitatively distinct molecular subtypes relying on the expression of 33 core genes. (**Figures S1C, S1D**). Ultimately, we identified two distinct clusters, including 270 cases in depression cluster 1 and 360 cases in depression cluster 2, termed these clusters D. cluster1 and D. cluster2. Further analysis demonstrated that the expression of 33 genes was remarkably different in the above two molecular subtypes (**Figures 2D**). We then carried out GSVA enrichment analysis based on the “H: hallmark gene sets” to investigate the underlying molecular biological alterations that are associated with the two unique depression-related IBD molecular subtypes. GSVA results showed that D. cluster2 was considerably enriched in processes associated with the stimulation of inflammation, including interferon-gamma/alpha response, TNF-alpha signalling *via* NF-κB, IL-6 JAK-STAT3 signalling and inflammatory response. Nevertheless, D. cluster1 presented enrichment pathways prominently associated with metabolic-related pathways, such as the bile acid metabolism and fatty acid metabolism pathways (**Figure 2E**). In addition, the expression of pro-inflammatory-related signature factors (S100A8, S100A9, TNF-α, IL-1B, IL-6, IL-17, INF-γ) was upregulated in D. cluster2 at the mRNA level, while intestinal barrier function-related marker protein (LGR5, MUC2) was down-regulated. In addition, IL-22, an inflammation-associated cytokine is also upregulated in D. cluster2, but its role in inflammation remains controversial (**Figures 2F, G**). We consequently speculate that the patients of D. cluster2 might have a worse prognosis due to inflammatory activation and intestinal barrier dysfunction.

### Depression-related molecular subtypes characterized by distinct immune and metabolism landscapes

**Figure 3:**
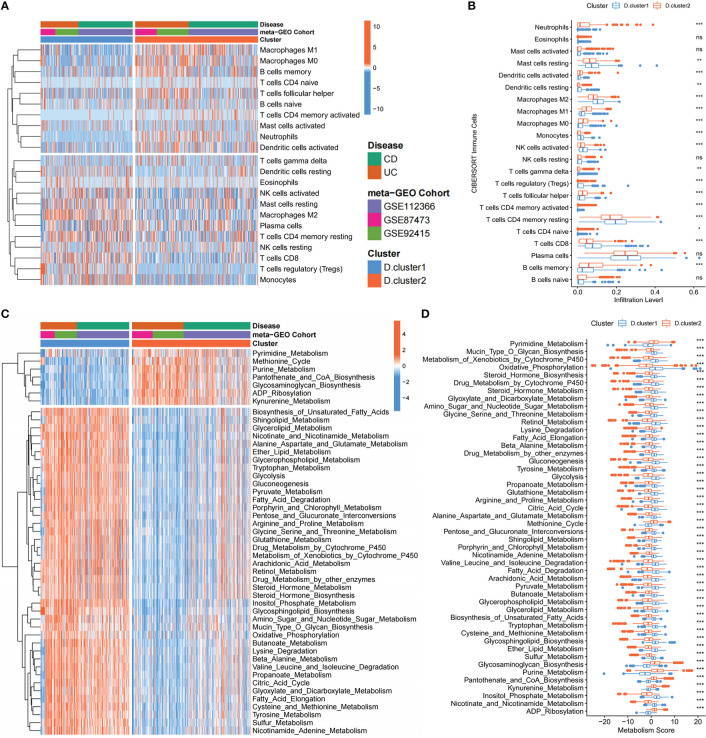
Depression-related molecular subtypes characterized by distinct immune and metabolism landscapes. **(A, B)** Heatmap and boxplot show the abundance of 22 immune infiltrating cells in two depression-related molecular subtypes. The upper and lower ends of the boxes represented the interquartile range of values. The lines in the boxes represented the median value, and the black dots showed outliers. **(C, D)** Heatmap and boxplot show the metabolic mapping in two depression-related molecular subtypes. The upper and lower ends of the boxes represented the interquartile range of values. The lines in the boxes represented the median value, and the black dots showed outliers. The asterisks represented the statistical p-value (ns P > 0.05; *P < 0.05; **P < 0.01; ***P < 0.001).

Given that immune manipulation plays an indispensable role in IBD, we constructed a heatmap and boxplot with CIBERSORT (20), a deconvolution approach based on support vector regression for classifying subsets of immune cells, to visualize and compare the relative abundances of 22 immune infiltrating cell subpopulations among distinct Depression-related molecular subtypes in IBD patients (**Figures 3A, B**). Pro-inflammatory immune cells, such as Neutrophils and macrophage M1, were mainly enriched in the D. cluster2, while macrophages M2 and regulatory T (Treg) cells, which embrace anti-inflammatory effects, were markedly elevated in the D. cluster1 subtype. We further investigated the specific association between every 33 genes and immune cell infiltration by using Spearman’s correlation analyses (**Figure S2A**). High expression of BTN3A1, OLFM4, FADS2, LRFN5 and PAX5 was significantly associated with pro-inflammatory immune cells (Neutrophils or macrophages M1), whereas RERE, CDSN, FH, FHIT, HIST1H3H, PCLO, SEMA6D, SYNE2 and ZSCAN16 expression exhibited a positive correlation with the anti-inflammatory, immune response (macrophages M2 or regulatory T cells). The above results indicated that the variation in immune infiltration between subtypes resulted from differences in the expression of 33 core genes.

Moreover, we also explored the metabolic mapping differences between the two subtypes. We discovered that D. cluster2 downregulates a variety of metabolic pathways compared to D. cluster1, including glucose metabolism (Glycolysis, Gluconeogenesis, Citric Acid Cycle and Oxidative Phosphorylation), lipid metabolism (Shingo-lipid Metabolism, Glycerolipid Metabolism and Fatty Acid Elongation), amino acid metabolism (Alanine Aspartate and Glutamate Metabolism, Tryptophan Metabolism, Arginine and Proline Metabolism, Glycine Serine and Threonine Metabolism) and so on (**Figures 3C, D**). In particular, pathways related to metabolic homeostasis [Retinol Metabolism (28), Steroid Hormone Metabolism (29) and Nicotinate and Nicotinamide Metabolism (30)] were also downregulated. Therefore, D. cluster2 can be regarded as a hypometabolic subtype. Notably, kynurenine metabolism, which has been reported to contribute to the development of depression-like behavior (15), is significantly activated in D. cluster2. Given the above characteristics of the patients in D. cluster2, it is hypothesized that those patients may suffer from depression and inflammatory bowel disease simultaneously. To validate those hypotheses, we compared the expression levels of previously published depression-related factors (31) between the two subtypes, and we observed those genes that were upregulated in depression were also upregulated in D. cluster2, compared to D. cluster1 (**Figure S2B**).

### Construction of the Depression score (D. score) of IBD and investigation of its clinical significance

**Figure 4:**
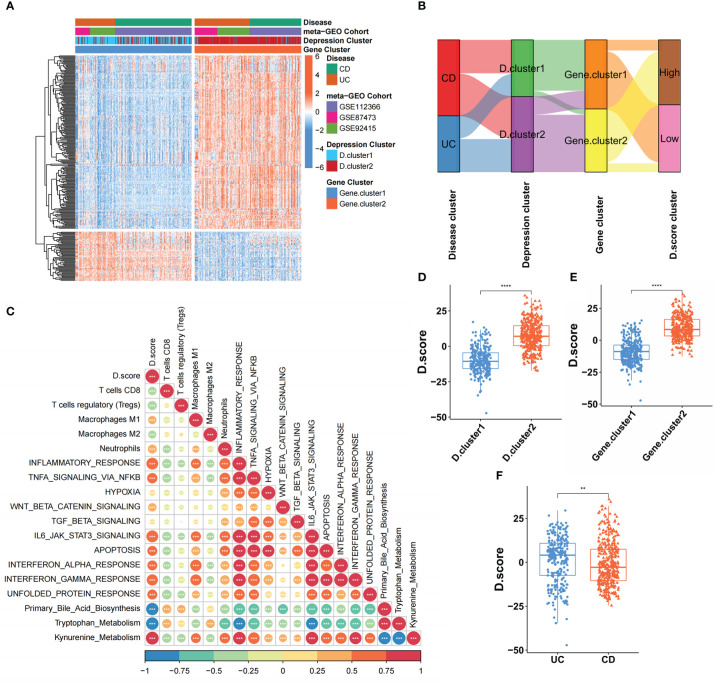
Construction of the Depression score (D. score) of IBD and exploration of its clinical relevance. **(A)** Unsupervised clustering of DEGs among the two molecular subtypes to classify patients into different genomic subtypes termed as the gene. cluster 1 and gene. cluster 2 respectively. The gene signature subtypes, **(D)** clusters, and disease subtypes were used as patient annotations. **(B)** Alluvial diagram showing the changes of D.clusters, disease subtypes, gene clusters and D.score. **(C)** Correlations between D.score and the known gene signatures in meta-cohort using Spearman analysis. A negative correlation was marked with blue and a positive correlation with orange. **(D)** Differences in D.score between two **(D)** clusters in GEO meta-cohort. **(E)** Differences in D.score between two gene clusters in GEO meta-cohort. **(F)** Differences in D.score between two disease subtypes in GEO meta-cohort. The asterisks represented the statistical p-value (**P < 0.01; ****P < 0.0001).

Even though IBD patients were split into two molecular categories using a consensus clustering technique based on the expression of 33 core genes, the underlying genetic alterations and expression perturbations within these subtypes were unclear. In light of these questions, we further aim to investigate the possibility of the difference in the transcriptional expression of 33 key genes between the two molecular subtypes of inflammatory bowel disease. Two hundred sixty-three differentially expressed genes (DEGs) were identified as hallmark genes associated with depression in individuals with IBD. Analysis of these signature genes using GO enrichment revealed that immune-related biological processes, regulation of response to stimulus, and inflammatory response were significantly over-represented (**Figure 3SA**). These findings added to the evidence that DEGs were inflammatory and immune-related, making them a potential “gene signature” for IBD-related depression. Based on the 263 most representative depression phenotype-related signature genes in IBD patients, we performed an unsupervised consensus clustering analysis and obtained two stable transcriptomic phenotypes (**Figures S3B, C**). These stratifications separated patients into two distinct depression gene signature groupings with unique biological characteristics, denoted as the gene. cluster1 and gene. cluster2. (**Figure 4A**).

Although our results categorized IBD patients into two subgroups based on the 33 core genes, our analyses were limited to the patient population and were unable to accurately predict the depressive state of individual IBD patients. Therefore, we developed a scoring scheme termed the Depression score (D. score), which is based on the depression-related signature genes, to quantify the states of individual IBD patients. To better illustrate the characteristics of IBD-related depression gene signature, we also used the Spearman analysis to examine the correlation between known biological characteristics and the D. score (**Figure 4C**). The correlation matrix’s heatmap revealed that the D. score was significantly positively linked with kynurenine metabolism., immune activation process and inflammatory response but negatively correlated with primary bile acid biosynthesis and Immunosuppressive-relevant signatures. The alluvial diagram was used to visualize the attribute changes of individual patients (**Figure 4B**). These results indicated that D. cluster2 and gene. cluster2 was linked to a higher D. score, whereas D. cluster1 and gene. cluster1 exhibited a lower D. score (**Figures 4D, E**). In addition, we found that UC patients had a higher D. score than CD patients (**Figure 4F**), which means UC patients were more likely to experience depression.

### The role of the D. score in assessing intestinal inflammation and predicting anti-TNF-alpha benefits

**Figure 5:**
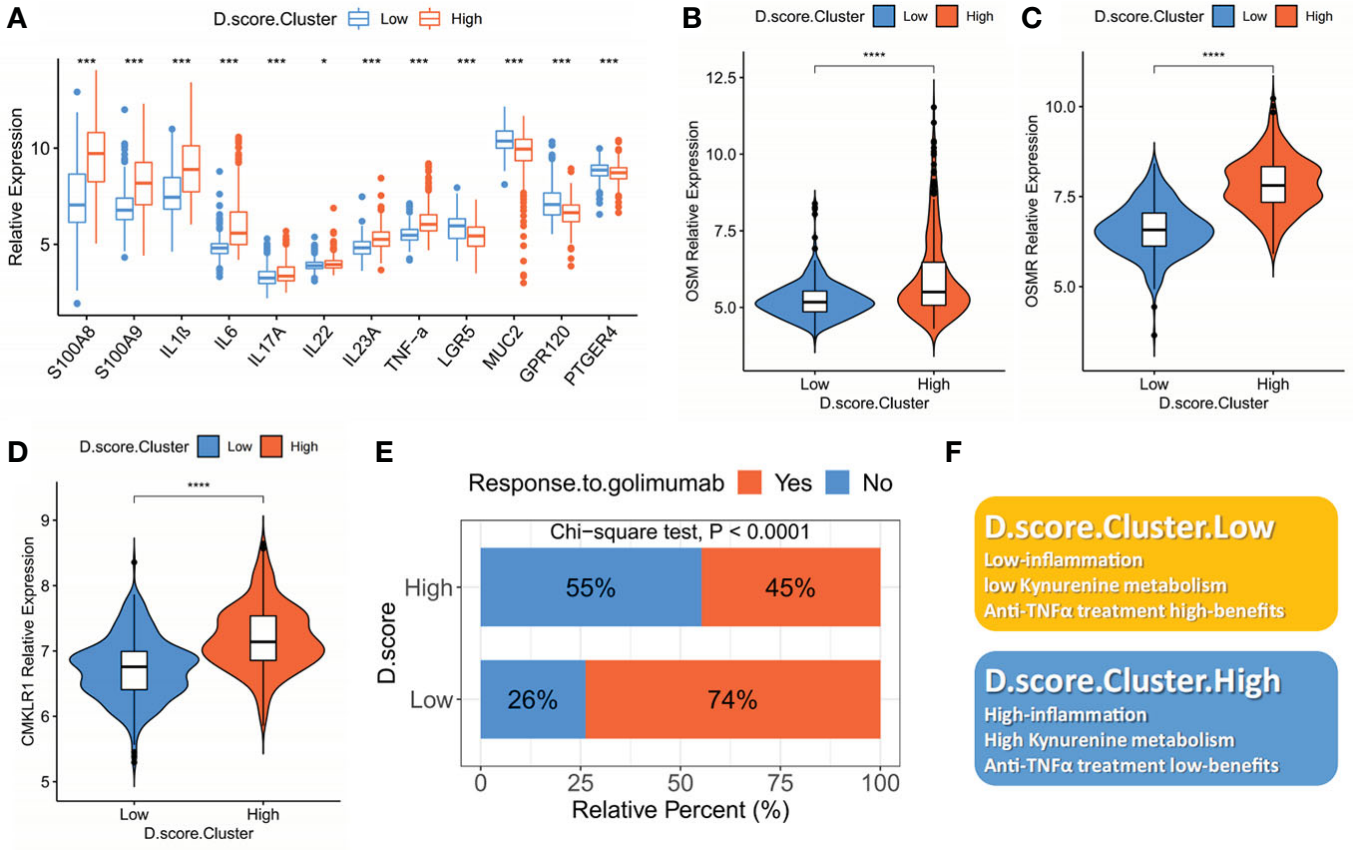
The role of the D. score in assessing intestinal inflammation and predicting anti-TNF-alpha benefits. **(A)** The mRNA expression levels of intestinal inflammatory markers (S100A8, S100A9, TNF-α, IL-1B, IL-6, IL-17, IL-22, INF-γ) and barrier function markers (LGR5, MUC2) in high D. score versus low D. score subgroups. **(B)**, **(C)** and **(D)** The mRNA expression levels of anti-TNF-alpha-resistant biomarkers (CMKLR1, OSM and OSMR) in high D. score versus low D. score subgroups. The asterisks represented the statistical P-value (*P < 0.01; ***P < 0.001; ****P < 0.0001). **(E)** The fraction of patients with clinical response to anti-TNF-alpha immunotherapy (golimumab) in low or high D. score groups (Chi-square test; p<0.0001). **(F)** Summary diagram of the biological characteristics in low or high D. score groups.

Monoclonal antibody biotherapy (anti-TNF) is a breakthrough therapy for IBD, yet 30% of patients do not react, and some even develop drug resistance (32–34). We used the D. score to assess IBD patients’ intestinal inflammation by comparing inflammatory factors and gut barrier integrity-associated proteins at the mRNA level. As expected, proinflammatory-related signature factors (S100A8, S100A9, TNF-α, IL-1B, IL-6, IL-17, IL-22) expression were upregulated in the high D. score cluster, while intestinal barrier function-related signature protein (LGR5, MUC2) was down-regulated. In other words, a lower D. score in IBD patients was significantly associated with better gut barrier function and lower intestinal inflammatory load (**Figure 5A**). Following the discovery of the D. score’s strong correlation with the immune-inflammatory response, we tested the hypothesis that the genetic signature of depression may be used to predict patients’ responses to anti-TNF-alpha therapies. OSM and OSMR are over-expressed in the great majority of active IBD lesions, especially in individuals whose condition is resistant to treatment with anti-TNF-alpha antibodies (35). Our research shows that patients with low D. scores have an obviously low expression of OSM and OSMR, which indicates a potential response to anti-TNF-alpha immunotherapy (**Figures 5B, C**). In addition, another possible biomarker and therapeutic target for IBD is CMKLR1 (also known as ChemR23), which is of significant relevance for anti-TNF-alpha-resistant patients (36). We also found that CMKLR1 was enriched in the high D. score cluster (). Furthermore, a consistent result has also been observed in our meta-cohort, lower D. scores in IBD patients were significantly associated with better clinical outcomes that the majority of patients responded to golimumab treatment and higher anti-TNF-alpha immunotherapies benefits (**Figure 5E**). In conclusion, our data provide compelling evidence that the D. score is related to the efficacy of anti-TNF-alpha immunotherapies and may be used to predict the outcome of IBD case scenarios. (**Figure 5F**).

### Antidepressants can resolve intestinal inflammation in a preclinical model of D. cluster2 patients

In Western nations, antidepressants are used by 10% to 30% of persons with IBD (37–39), and the systematic reviews (40–43) and a narrative review (44) that has been carried out to this point have shown that antidepressants have a positive influence on the well-being of patients who have IBD, however, the evidence supporting their involvement in the treatment of the condition is scant. We furthermore wanted to investigate whether antidepressants could alleviate intestinal inflammation while relieving mental health in IBD patients and explore the molecular mechanisms involved. Previous research established that mice given DSS had physiological alterations similar to IBD and depressive-like behaviours (45, 46). Thus, we consider DSS-treated mice to be a preclinical model for D. cluster2 patients who have both IBD and depression. Given that Selective Serotonin Reuptake Inhibitors (SSRIs) are the most widely used antidepressants nowadays, we selected two of them for the following experiment.

We then treated C57BL/6 mice with 3% DSS and gave them either paroxetine (PX) orally or fluoxetine (FX), two frequently used SSRIs, to see whether the drugs would have a therapeutic impact on colitis. (**Figure 1A**). Surprisingly, PX administration alleviated body weight loss (**Figure 1B**) and increased colon length (**Figures 1C, D**). The DAI score used to assess the severity of colitis symptoms was significantly lower in the DSS+PX group compared to the DSS group. (**Figure 1E**). Relieved disruption of glandular structures and crypt foci and reduced inflammatory cell infiltration in the colonic epithelium were observed in the DSS+PX group compared to the DSS group by H&E-stained (**Figures 1F, G**). Although FX also had a modest protective effect, disease indexes such as body weight, bowel length, and DAI scores showed no statistical significance. Collectively, PX can alleviate intestinal inflammation in a preclinical model of D. cluster2 patients and could be a potential medicine for the treatment of IBD.

### Paroxetine down-regulates multiple inflammatory pathways and alters host metabolism

**Figure 6:**
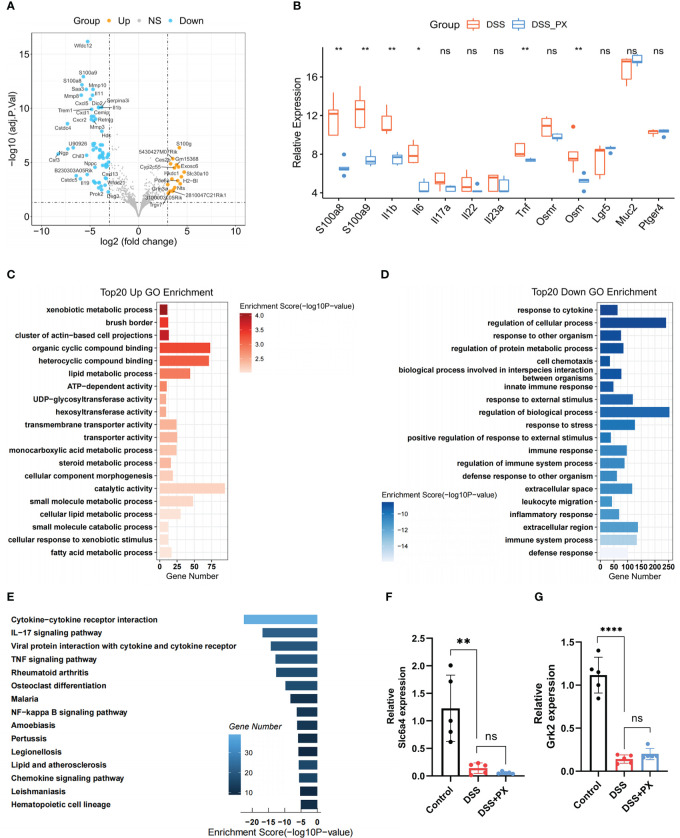
Paroxetine down-regulates multiple inflammatory pathways and alters host metabolism. **(A)** Volcano plot showing the differentially expressed genes between DSS+PX and DSS groups by RNA-seq. **(B)** Boxplot showing the mRNA expression levels of intestinal inflammatory markers (S100a8, S100a9, Tnf-α, Il-1b, Il-6, Il-17a, Il-22, Il23a, Ptger4), barrier function markers (Lgr5, Muc2), and anti-TNF-alpha-resistant biomarkers (Osm and Osmr) in DSS+PX versus DSS groups. The asterisks represented the statistical P-value. **(C)** GO enrichment analysis of top 20 upregulated genes in DSS+PX groups. **(D)** GO enrichment analysis of top 20 downregulated genes in DSS+PX groups. **(E)** KEGG analysis of downregulated DEGs between DSS+PX and DSS groups. **(F)** Quantitative PCR analysis of Slc6a4 expression in mice colon tissues of DSS+PX, DSS, and control groups. **(G)** Quantitative PCR analysis of Grk2 expression in mice colon tissues of DSS+PX, DSS, and control groups. Data are pooled from each independent experiment with n = 5 mice per group; The asterisks represented the statistical P-value (ns P > 0.05; *P < 0.05; **P < 0.01; ****P < 0.001).

To illuminate the underlying mechanisms of how PX remitted the colitis severity, we performed mRNA sequencing (RNA-seq) with mice colon (**Table S2**). Our results demonstrated that PX treatment in the DSS mice model down-regulated several factors of inflammation and chemokines (**Figures 6A, B**), especially several core pro-inflammatory factors (S100a8, S100a9, Tnf-α, Il-1β, Il-6). Besides, we found that paroxetine decreased the expression of OSM, implying that it might improve the effectiveness of anti-TNF-α therapy. Through GO and KEGG enrichment analysis, we discovered that paroxetine could mediate various biological processes to alleviate intestinal inflammation. On the one hand, PX down-regulates several inflammatory processes, such as response to cytokine, response to stress, inflammatory response, cytokine−cytokine receptor interaction, IL−17, TNF and NF−κB signalling pathway (**Figures 6D, E**). On the other hand, PX also upregulates several metabolic pathways that are missing in D. cluster2, which indirectly suggests that it can reverse specific metabolic pathways, for instance, steroid metabolic process and fatty acid metabolic process, and thereby alleviate intestinal inflammation (**Figure 6C**).

### Paroxetine alleviates intestinal inflammation by modulating the structure and function of gut microbiota

**Figure 7:**
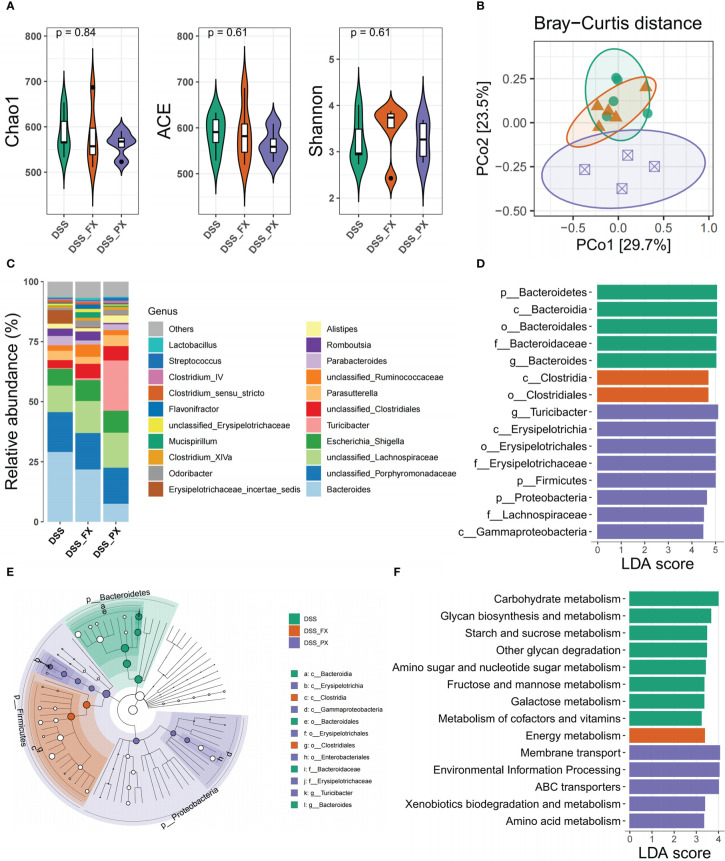
Paroxetine alleviates intestinal inflammation by modulating the structure and function of gut microbiota. **(A)** Alpha diversity includes Chao1, Ace, and Shannon indices. **(B)** Genus-level principal coordinate analysis (PCoA) plot based on Bray–Curtis distances. **(C)** Heatmap of the selected most differentially abundant features at the genus level. **(D)** LDA score plot of differentially abundant taxonomic features (LDA score for discriminative features>3). **(E)** Taxonomic cladogram produced from LEfSe analysis. **(F)** LDA score plot of differentially abundant of function prediction features (LDA score for discriminative features >3). Orange bars indicate taxa enrichment in DSS+FX groups, purple bars indicate taxa enrichment in DSS+PX groups and green bars indicate taxa enrichment in DSS groups.

Even though the above results have validated the anti-inflammatory phenotype of PX, its molecular mechanism remains to be further investigated. From the literature survey, we learned that PX has two targets (Slc6a4 and Grk2) in the intestine. However, the expression of the above targets decreased after DSS treatment, and their expression levels were not altered after both DSS and DSS +PX treatment, suggesting that the anti-inflammatory effect of PX is not exerted through the previous mechanism (**Figures 6F, G**). In our earlier conclusions, we found that PX significantly downregulated the S100a8 and S100a9 (**Figure 6B**), which are essential for gut microbiota development (47), so we hypothesized that paroxetine might exert its anti-inflammatory effects through gut microbes. We, therefore, performed a 16S ribosomal RNA analysis of mice feces. The alpha diversity found no statistical difference between DSS+PX and DSS groups (**Figure 7A**). Disparities in the organization of the gut microbiota were found between the DSS+PX and DSS groups, as shown by principal coordinate analysis (PCoA), which was based on Bray–Curtis metric distances. (**Figure 7B**).

Moreover, we analyzed the geography of the gut microbiota to provide light on the variations in the makeup of the gut flora. Firmicutes represented the richest phyla, followed by Proteobacteria in the DSS+PX group, while Bacteroidota enriched in the DSS group (**Figures 7C, D**). Compared with the DSS group, the DSS+PX group displayed differential biological compositions at the genus level (**Figure 7C**). LEfSe showed that treatment with paroxetine was dominated by members of Firmicutes (Turicibacter) and Proteobacteria members. In contrast, increased levels of Bacteroidota (Bacteroides) were instead observed in the DSS group (**Figures 7D, E**) (**Table S3**). The results of the microbial function prediction indicated that paroxetine could shift the microbial composition (**Figure 7F**) from Carbohydrate metabolism (glycan biosynthesis and metabolism, starch and sucrose metabolism, amino and nucleotide sugar metabolism, fructose and mannose metabolism, galactose metabolism) toward amino acid metabolism (membrane transport and ABC transporters).

## Discussion

In this study, we describe the transcriptional heterogeneity of 33 core genes that drive depression in IBD patients and use these key factors to molecularly classify patients into two subtypes with distinct immune and metabolic profiles, and identify D. cluster2 with features of IBD combined with depression.

Our research demonstrates that D. cluster1 can be regarded as a low-inflammatory subtype, characterized by an enrichment of anti-inflammatory immune cells, such as macrophages M2 and regulatory T cells (Treg), and metabolic pathways (Retinol Metabolism, Steroid Hormone Metabolism and Nicotinate and Nicotinamide Metabolism) and a reduction in pro-inflammatory cytokines. In contrast, D. cluster2 contains a bunch of pro-inflammatory immune cells (Neutrophils and macrophages M1), cytokines and metabolic pathways, such as kynurenine metabolism associated with both intestinal inflammation and depression, corresponding to the high-inflammatory subtype. The above results indicate that as depression-related symptoms occur in people with IBD, their intestinal inflammation becomes more aggressive, which undoubtedly exacerbates an existing condition. Thus, it explains why IBD patients with symptoms of depression were more likely to have a greater risk of flare-ups, need a higher dose of medication, be admitted to the hospital, visit the emergency room, or have surgery (48).

Moreover, we found that intestinal barrier function-related marker protein (LGR5, MUC2) was down-regulated in D. cluster2 compared to D. cluster1. On the one hand, these results could prove that depression combined with IBD accelerates the disruption of intestinal barrier function and aggravates gut inflammation. On the other hand, disruption of the intestinal barrier will lead to disruption of the gut vascular barrier (GVB), which is similar to the blood-brain barrier (BBB) and connects the bowel to the liver. Non-closure of the GVB allows inflammation to spread to distant organs, such as our brain, which can lead to mental deficiencies (26). This resounding evidence explains how intestinal inflammation contributes to the development of depression.

Further, to shed light on the association between depression and molecular subtypes of IBD, a scoring system was established to comprehensively assess enteric inflammation in individuals with IBD. Our research demonstrates that the higher D. score in IBD patients was significantly associated with worse gut barrier function and higher intestinal inflammatory load, which suggests that the D. score provides a reliable indicator of the inflammatory condition of the gut in IBD. Our data also revealed a markedly negative correlation between D. score and anti-TNF-alpha biotherapeutic benefits. Consistent with previous studies, IBD patients with depression were at increased risk of nonresponse to biological therapy (49). This recommended D. score served as a viable and stable instrument for the thorough evaluation of individual responses to anti-TNF-alpha biotherapeutics.

Considering that many anti-inflammatory medicines also have anti-depressive and anti-anxiety properties, the use of antidepressants as adjuvant treatment in IBD has been highlighted (17). Although antidepressants have been used in the treatment of colitis in previous studies (50, 51), the anti-inflammatory mechanisms have not been thoroughly investigated. Subsequently, in clinical practice, we selected the two most widely used antidepressants for animal experiments and uncovered that paroxetine could resolve intestinal inflammation in a preclinical model of UC with depressive symptoms, whereas fluoxetine does not. This is consistent with the results of a single randomized, double-blind study involving 26 people with IBD, in which fluoxetine was shown to be no more effective than a placebo (52). Through high-throughput sequencing technology, we also show that paroxetine can inhibit the expression of multiple inflammatory factors at the mRNA level and down-regulate multiple inflammation-related pathways, thus explaining the mechanism of paroxetine anti-inflammation at the molecular level.

Gut bacteria protect the intestinal epithelium under normal physiological circumstances (53). When intestinal flora is disturbed, a wide range of bacteria and inflammatory chemicals may compromise the integrity and permeability of tight junctions, hence compromising the gut mucosa’s capacity to operate as a barrier (54). Environmental disturbances and gut barrier dysfunction frequently enhance vulnerability to inflammatory-related conditions. Considering that the gut microbiome plays a crucial role in the etiology of IBD (55), the investigation into whether or if the gut flora contributes to the protective effect of paroxetine on colitis is warranted. In addition, the last decade has witnessed an exponentially growing interest in gut microbiota and the gut-brain axis in health and disease. Accumulating evidence from preclinical and clinical research indicates that gut microbiota, and their associated microbiomes, may influence pathogenic processes and thus the onset and progression of various diseases, including neurological and psychiatric disorders. The hormones and neurotransmitters produced by the nervous system may have a modulating effect on immune function as well as metabolic function in the gut through the regulation of gut microorganisms (56, 57). Therefore, as a disorder of the brain-gut axis, intestinal flora may play an important role in the pathogenesis of IBD patients with depression.

Here, the PCoA results showed that there were differences in biological community structure between the DSS and DSS+PX groups, even though our alpha diversity results revealed no significant difference. Multiple studies have shown that the phylum Firmicutes is often decreased in proportionate abundance in the feces of IBD patients compared to healthy persons, but the phylum Proteobacteria, including Enterobacteriaceae and Escherichia coli, is frequently increased (58). Our study shows that paroxetine treatment of mice with colitis can modulate gut dysbiosis and reverse the alters described above according to LEfSe analysis. In other words, paroxetine elicited gastroprotective effects in mice maybe by increasing the growth of Turicibacter (59), a beneficial gut bacterium, and by decreasing populations of pathogenic Bacteroides. The results of the microbial function prediction indicated that paroxetine could shift the microbial composition from Carbohydrate metabolism toward Amino acid metabolism. It is demonstrated that paroxetine not only alters the composition of intestinal microorganisms but also systematically transforms their functional mapping, thereby alleviating intestinal inflammation by rescuing intestinal microecology.

There may be some possible limitations in this study (1). The use of paroxetine to treat DSS-treated mice is prophylactic rather than therapeutic, and exploring its therapeutic value in subsequent experiments will further increase its clinical transformational implications (2). We did not investigate in an in-depth manner the long-term therapeutic implications of paroxetine, improving this scientific question in subsequent studies will contribute to better understanding of the role of antidepressants in the treatment of IBD.

## Conclusions

Our study revealed for the first time to our knowledge a group of IBD patients with depression by molecular typing and illustrated their immunological and metabolic profile in detail. Quantitatively evaluating the IBD-related depression gene signature of individual patients will promote more effective immunotherapy strategies and antidepressant paroxetine may help reduce intestinal inflammation.

## Data availability statement

The data presented in the study are deposited in the NIH National Center for Biotechnology Information Sequence Read Archive (SRA) repository and accession numbers can be found below: https://www.ncbi.nlm.nih.gov/bioproject/PRJNA904496; https://www.ncbi.nlm.nih.gov/bioproject/PRJNA903959.

## Ethics statement

The animal study was reviewed and approved by Ethics Committee of Shanghai Renji Hospital.

## Author contributions

ZW, JH, and XL conceptualized and supervised the study; ZW, XL, and JH acquired funding; LN, XW, YM, TT, and BX acquired and analyzed the data; LN, XW, YY, and ZG performed the investigation and experiments; LN, XW, ZC, and BX drafted the manuscript; ZW, HC, XL, and JH reviewed and edited the manuscript. All authors contributed to the article and approved the submitted version.

## Supplementary material

Supplementary Figure 1 Unsupervised clustering of two molecular subtypes by 33 core genes in the meta-GEO cohort **(A)** The interaction of expression of 33 core genes in IBD. **(B)** Bar plot shows the GSVA score of representative Hallmark pathways curated from MSigDB in distinct molecular subtypes. **(C)** The relationship between cophenetic, dispersion, residuals, and silhouette coefficients with respect to the number of clusters. **(D)** Heatmap representation of NMF clustering for 33 core genes in the meta-GEO cohort with cluster numbers from 2 to 10.

Supplementary Figure 2 Depression-related molecular subtypes characterized by distinct immune and metabolism landscapes **(A)** Heatmap of the correlation between 33 core genes and 22 immune infiltrating cells. **(B)** The expression levels of depression-related factors between the two subtypes. The asterisks represented the statistical P-value (*P < 0.05; **P < 0.01; ***P < 0.001; ****P < 0.0001).

Supplementary Figure 3 Unsupervised clustering of 263 most representative depression phenotype-related signature genes in the IBD cohort. **(A)** Functional annotation for 263 most representative depression phenotype-related signature genes using GO enrichment analysis. **(B)** The relationship between cophenetic, dispersion, residuals, and silhouette coefficients with respect to the number of clusters. **(C)** Heatmap representation of NMF clustering for 263 most representative depression phenotype-related signature genes in meta-GEO with cluster numbers from 2 to 10.
