# Supplementary figures and images for: Identification and investigation of depression-related molecular subtypes in inflammatory bowel disease and the anti-inflammatory mechanisms of paroxetine
Source: Front Immunol. 2023 Feb 27;14:1145070. doi: 10.3389/fimmu.2023.1145070 (PMC10008943; doi:10.3389/fimmu.2023.1145070)

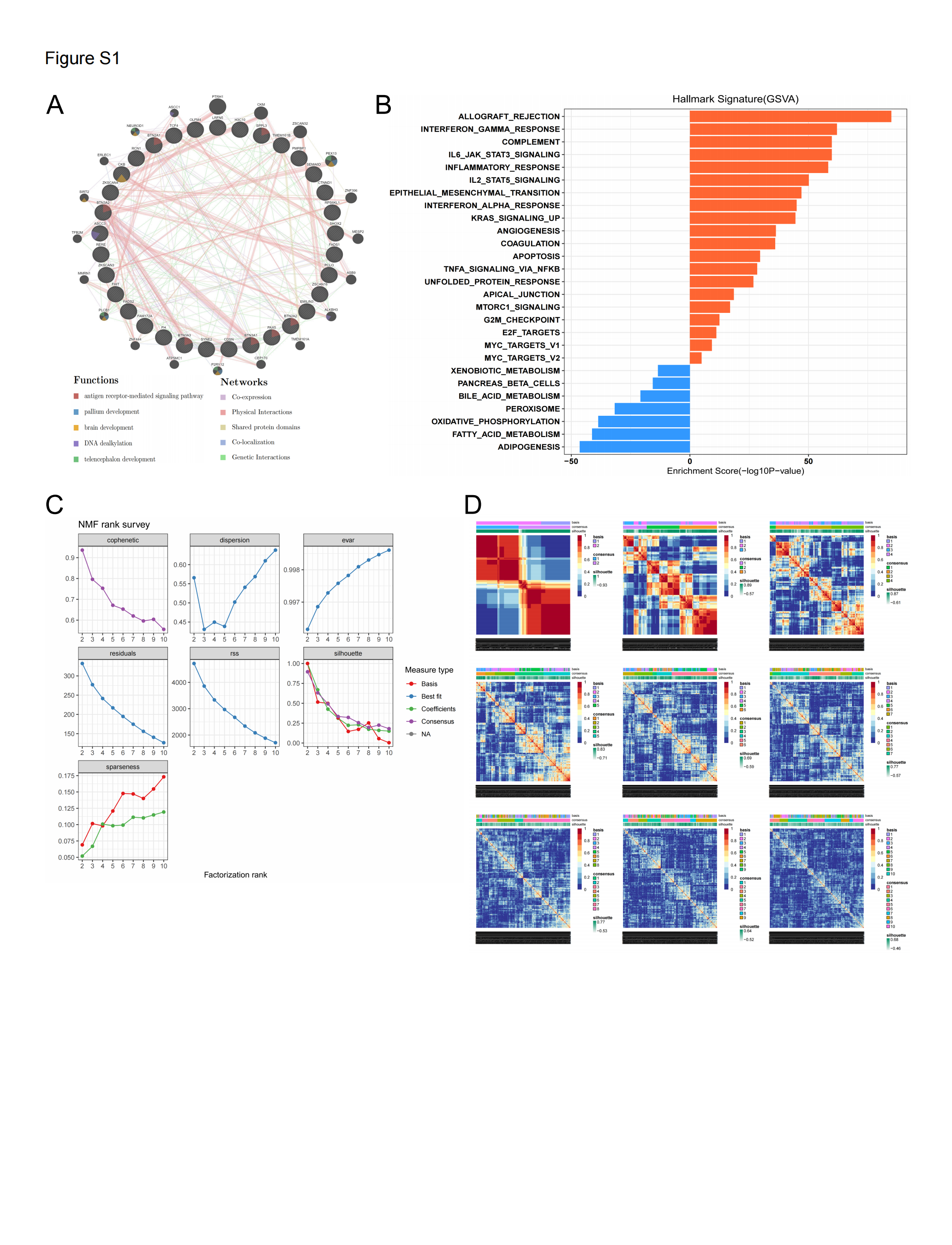

Supplement: Supplementary Figure 1 — Unsupervised clustering of two molecular subtypes by 33 core genes in the meta-GEO cohort (A) The interaction of expression of 33 core genes in IBD. (B) Bar plot shows the GSVA score of representative Hallmark pathways curated from MSigDB in distinct molecular subtypes. (C) The relationship between cophenetic, dispersion, residuals, and silhouette coefficients with respect to the number of clusters. (D) Heatmap representation of NMF clustering for 33 core genes in the meta-GEO cohort with cluster numbers from 2 to 10. [file Image_1.tif]

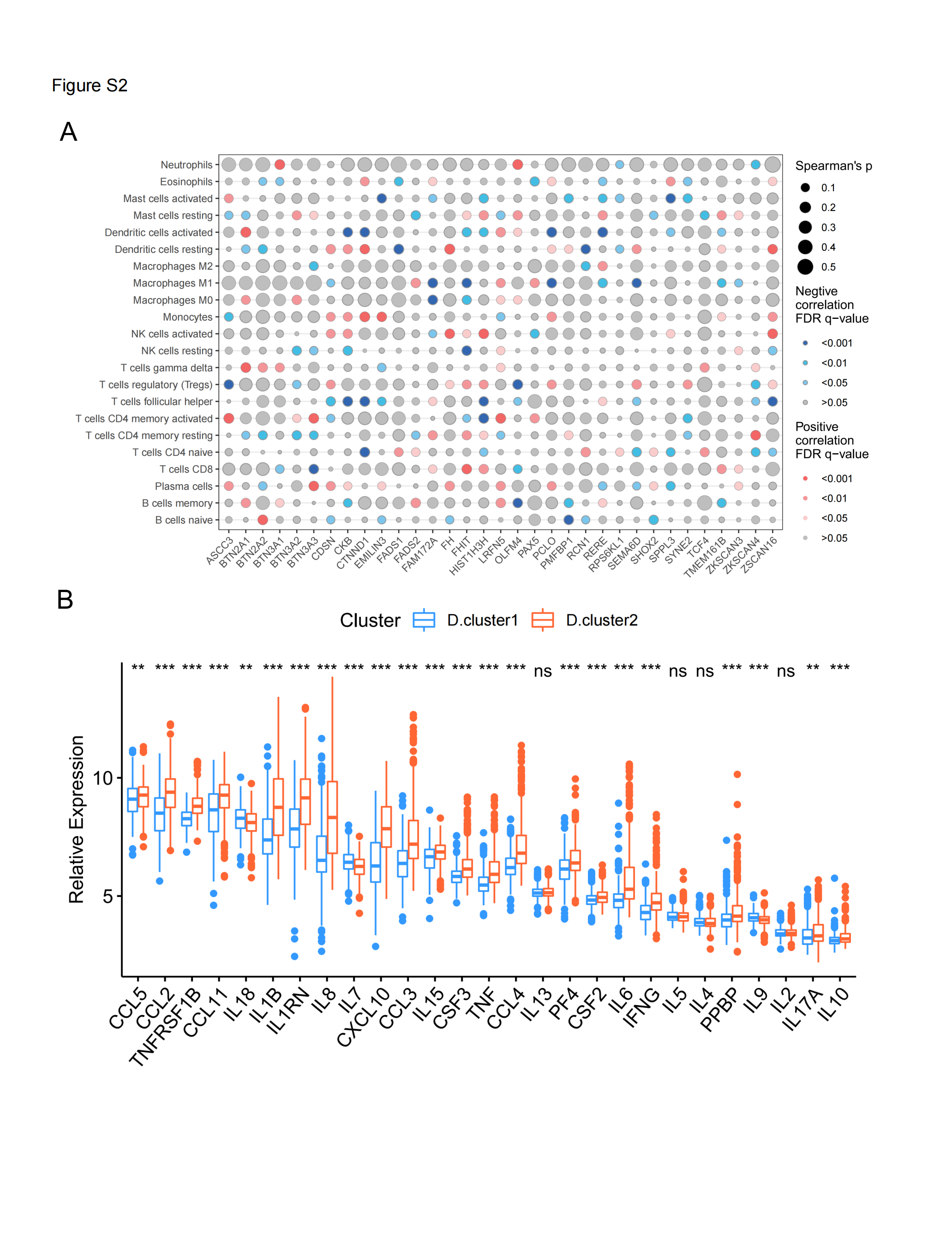

Supplement: Supplementary Figure 2 — Depression-related molecular subtypes characterized by distinct immune and metabolism landscapes (A) Heatmap of the correlation between 33 core genes and 22 immune infiltrating cells. (B) The expression levels of depression-related factors between the two subtypes. The asterisks represented the statistical P-value (*P < 0.05; **P < 0.01; ***P < 0.001; ****P < 0.0001). [file Image_2.tif]

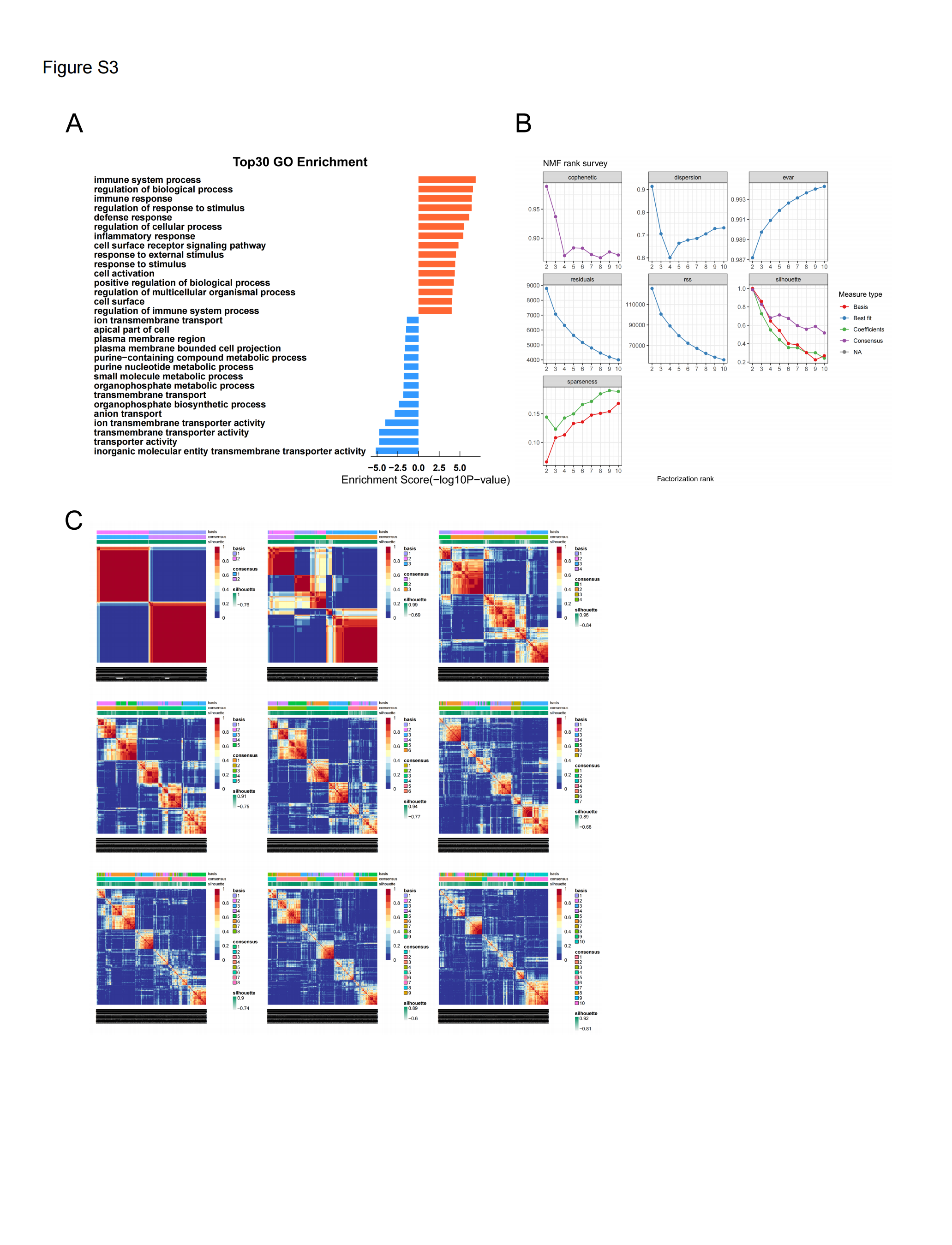

Supplement: Supplementary Figure 3 — Unsupervised clustering of 263 most representative depression phenotype-related signature genes in the IBD cohort. (A) Functional annotation for 263 most representative depression phenotype-related signature genes using GO enrichment analysis. (B) The relationship between cophenetic, dispersion, residuals, and silhouette coefficients with respect to the number of clusters. (C) Heatmap representation of NMF clustering for 263 most representative depression phenotype-related signature genes in meta-GEO with cluster numbers from 2 to 10. [file Image_3.tif]
